# Distinct genetic differentiation and species diversification within two marine nematodes with different habitat preference in Antarctic sediments

**DOI:** 10.1186/s12862-017-0968-1

**Published:** 2017-05-30

**Authors:** Freija Hauquier, Frederik Leliaert, Annelien Rigaux, Sofie Derycke, Ann Vanreusel

**Affiliations:** 1 Marine Biology Research Group, Biology Department, Ghent University, Krijgslaan 281, 9000 Ghent, Belgium; 2 Botanic Garden Meise, Nieuwelaan 38, 1860 Meise, Belgium; 3 Operational Directorate Taxonomy and Phylogeny, Royal Belgian Institute of Natural Sciences (RBINS), Rue Vautier 29, 1000 Brussels, Belgium

**Keywords:** Antarctica, Continental shelf, Cryptic species, *Desmodora*, Dispersal, Nematoda, Phylogeny, Population genetics, *Sabatieria*

## Abstract

**Background:**

Dispersal ability, population genetic structure and species divergence in marine nematodes are still poorly understood, especially in remote areas such as the Southern Ocean. We investigated genetic differentiation of species and populations of the free-living endobenthic nematode genera *Sabatieria* and *Desmodora* using nuclear 18S rDNA, internal transcribed spacer (ITS) rDNA, and mitochondrial cytochrome oxidase I (COI) gene sequences. Specimens were collected at continental shelf depths (200–500 m) near the Antarctic Peninsula, Scotia Arc and eastern side of the Weddell Sea. The two nematode genera co-occurred at all sampled locations, but with different vertical distribution in the sediment. A combination of phylogenetic (GMYC, Bayesian Inference, Maximum Likelihood) and population genetic (AMOVA) analyses were used for species delimitation and assessment of gene flow between sampling locations.

**Results:**

Sequence analyses resulted in the delimitation of four divergent species lineages in *Sabatieria*, two of which could not be discriminated morphologically and most likely constitute cryptic species. Two species were recognised in *Desmodora*, one of which showed large intraspecific morphological variation. Both genera comprised species that were restricted to one side of the Weddell Sea and species that were widely spread across it. Population genetic structuring was highly significant and more pronounced in the deeper sediment-dwelling *Sabatieria* species, which are generally less prone to resuspension and passive dispersal in the water column than surface *Desmodora* species.

**Conclusions:**

Our results indicate that gene flow is restricted at large geographic distance in the Southern Ocean, which casts doubt on the efficiency of the Weddell gyre and Antarctic Circumpolar Current in facilitating circum-Antarctic nematode species distributions. We also show that genetic structuring and cryptic speciation can be very different in nematode species isolated from the same geographic area, but with different habitat preferences (surface versus deeper sediment layers).

**Electronic supplementary material:**

The online version of this article (doi:10.1186/s12862-017-0968-1) contains supplementary material, which is available to authorized users.

## Background

Marine nematodes are the most abundant metazoan inhabitants of seafloor sediments and estimates of total marine species numbers (including parasites) are believed to exceed 50,000 [1]. Yet most of this diversity remains undescribed due to the difficult and time-consuming taxonomy, and logistically challenging sampling in remote (e.g., deep-sea) environments [2, 3]. To date, the number of described nematode species in the marine environment is ca. 12,000 (of which 6900 are free-living; [1]), which obviously covers only a limited fraction of total estimates [4, 5]. As a consequence, accurate characterisation of species diversity and biogeographic distributions for this highly abundant phylum is currently lacking and the study of macroecological patterns is inevitably limited to genus-level data. Additionally, the observation of extensive cryptic species diversity in species with different life history traits [6–11] further hampers correct estimation of global and local species diversity. Globally distributed nematode species may in fact constitute a series of cryptic species with more restricted geographic distribution for which morphological differences are not readily observable [7]. Coexistence of such cryptic nematode species at local scales may then partly be driven by differential ecological tolerances, preferences for abiotic factors and/or resource differentiation [12–14]. A profound understanding of species-specific preferences and life history traits (e.g., habitat preference, dispersal ability), in combination with knowledge on physical drivers of connectivity among marine populations (e.g., hydrodynamic forces, habitat characteristics) is thus imperative in the study of nematode species distribution patterns across various spatial scales and habitats.

The marine environment presents few obvious barriers to gene flow, and this has led to predictions of little genetic structure of marine species over large spatial scales [15], and speciation being mainly driven by broad-scale allopatric processes (e.g., [16, 17]). Alternatively, speciation in the sea can proceed without absolute barriers to gene flow along ecological boundaries, when divergent selection overwhelms the homogenising effect of gene flow (sympatric speciation; [18, 19]). In the Southern Ocean, genetic exchange between locations around the continent may be facilitated by the eastward Antarctic Circumpolar Current (ACC) and westward Antarctic Coastal Current (ACoC) systems, as well as the Weddell gyre [20–22]. Several Antarctic marine benthic invertebrates indeed have circum-Antarctic and eurybathic distributions (e.g., [23, 24]), reflecting a long history of isolation from other water masses and migrations up and down the slope during glacial cycles [22, 25]. However, evidence from DNA markers showed that their populations present substantial genetic differentiation and may be isolated over smaller spatial scales and depth ranges than previously thought [25, 26].

**Fig. 1:**
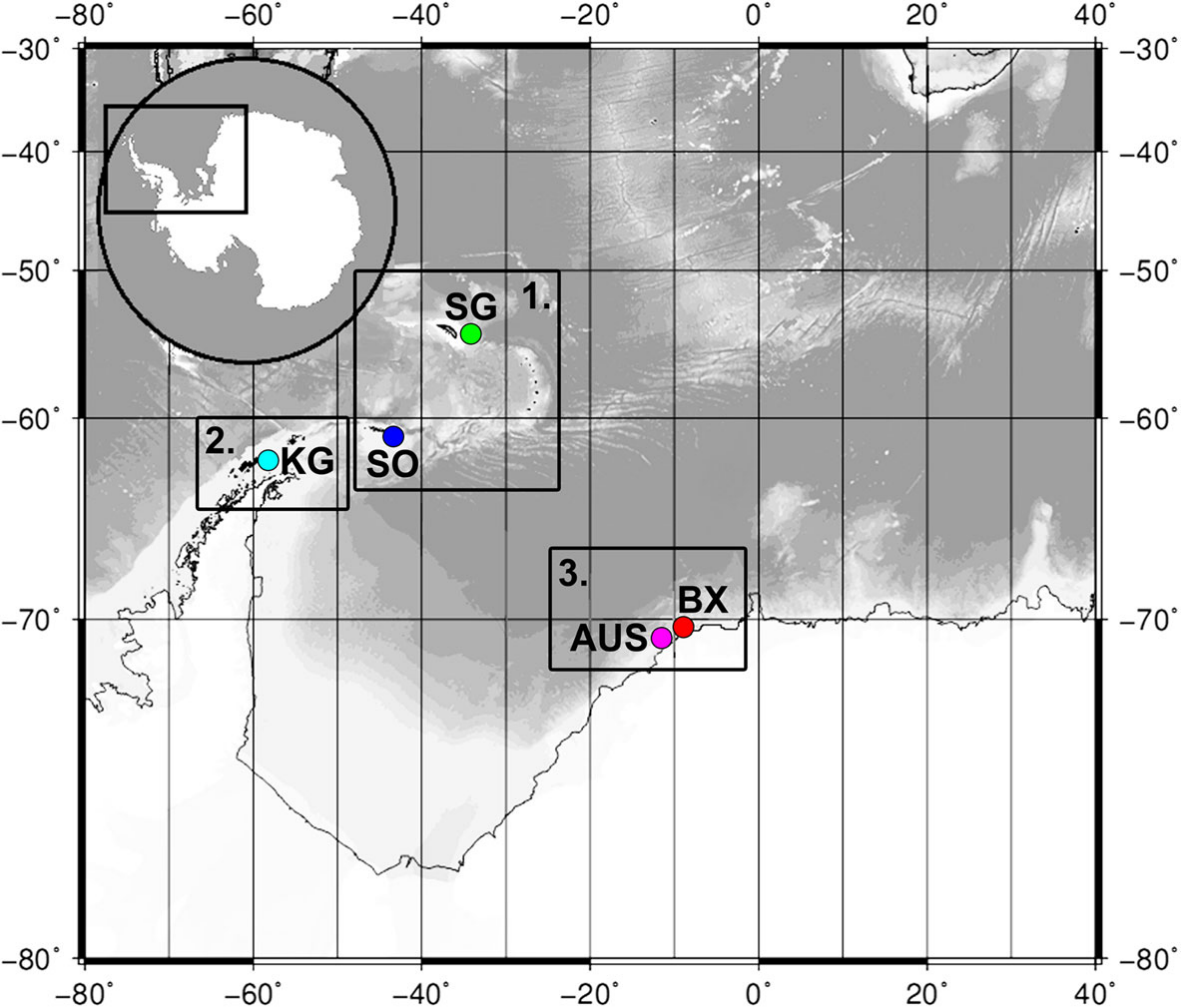
Map of Antarctica highlighting the geographic location of the five sampling stations. Box 1: Scotia Sea: SG = South Georgia, SO = South Orkneys; Box 2: Antarctic Peninsula: KG = King George; Box 3: eastern Weddell Sea: AUS = off Auståsen, BX = Bendex. The same colour code is maintained in figures and graphs throughout the manuscript. Adapted from cruise plot ANT-XXVII/3 [41] © Alfred Wegener Institute

In this study, we investigate the phylogeographic and population genetic structure of species from two marine nematode genera (*Sabatieria* Rouville, 1903 and *Desmodora* de Man, 1889) in the Antarctic using mitochondrial (cytochrome *c* oxidase subunit 1, COI) and nuclear (internal transcribed spacer (ITS) rDNA and small subunit (18S) rDNA) markers. Both types of molecular markers have been successfully applied in previous phylogenetic and population genetic studies of free-living nematodes (e.g., [2, 3, 7, 8, 27–29]), and – in absence of more variable alternatives – continue to serve as molecular markers for nematodes [30]. Spatial scale ranged from a few kilometres to >2000 km, comprising five locations at shelf depths spread along the Scotia Arc, Antarctic Peninsula and Weddell Sea (Fig. 1). The two genera are abundant and cosmopolitan in marine environments and have more than 100 described species each [31]. Four *Desmodora* and 15 *Sabatieria* species have been reported in the Antarctic [31–33]. *Desmodora* is a genus of epistratum-feeders (sensu [34]) that is often present in surface sediments, whereas *Sabatieria* species are deposit-feeders that typically reside in deeper sediment layers but are able to migrate upwards to access food and oxygen [32, 35]. Also in our study area, *Desmodora* and *Sabatieria* predominantly (but not exclusively) occurred at different sediment depths. This vertical segregation may have important consequences for dispersal since endobenthic marine nematodes do not possess a pelagic larval stage and are largely dependent upon passive transportation of individuals for their long-distance dispersal [11, 36]. Therefore, differential vertical distribution and abundance in the sediment is expected to influence their presence in the water column and the level to which they are prone to resuspension and passive dispersal via bottom currents [37, 38].

In light of current knowledge on cryptic speciation, cosmopolitan distribution and genetic structure in nematodes we expected to find 1/cryptic nematode species and strong genetic structuring in view of the large geographic distances between locations; 2/increased population genetic structure with increasing geographic distance (cf. isolation-by-distance principle IBD; [39, 40]), given the presumed limited dispersal capacity for nematodes (see also [11]); 3/ stronger population genetic structuring in *Sabatieria* than in *Desmodora* based on its preference for deeper sediment depths, assuming that surface dwellers have higher dispersal probability than species that occur deeper in the sediment.

## Methods

### Nematode collection, isolation and vouchering

Nematode specimens were collected onboard the German RV *Polarstern* in February–March 2011 (expedition ANT-XXVII/3, [41]) using a multicorer (MUC) device for undisturbed seafloor sampling. Five locations were sampled along the Scotia Arc (South Georgia SG, South Orkneys SO), Antarctic Peninsula (King George Island KG) and eastern Weddell Sea (Austasen AUS, Bendex BX; Fig. 1; Table 1), at shelf depths ranging between 240 and 440 m. Minimum distance between sampling locations was 15 km (AUS & BX), whereas the largest distance (as the crow flies) was almost 2300 km (KG & BX). MUC cores were divided into a surface (0–3 cm) and subsurface (3–5 cm) sediment slice. Samples were stored on a solution containing dimethyl sulphoxide, disodium EDTA, and saturated NaCl (abbreviated here as DESS; [42]) until further analysis in the lab. Nematodes were extracted from the sediments using 32 and 1000 μm sieves and density gradient centrifugation (Ludox specific density 1.18 g cm^−3^, centrifugation 3 × 12 min at 3000 rpm; [43, 44]).

**Table 1:** Overview of the five sampling locations, sampled specimens and number of sequences for species of both genera

				*SABATIERIA*					*DESMODORA*		
Location acronym	Latitude (Dm)	Longitude (Dm)	Depth (m)	species I	species II	species III	species IV	total per location	species I	species II	total per location
SG	54°25.612′S	35°41.799′W	257	114 | 10 | 2	25 | 4 | -	8 | 5 | -	- | - | -	147 | 19 | 2	17 | - | 9	- | - | -	17 | - | 9
SO	61°08.658′S	43°58.002′W	382	8 | - | -	25 | 3 | -	19 | 4 | -	- | - | -	52 | 7 | -	5 | - | 8	- | - | 12	5 | - | 20
KG	62°13.283′S	58°50.948′W	242	27 | 4 | -	1 | - | -	8 | 1 | -	- | - | -	36 | 5 | -	- | - | -	- | - | -	- | - | -
AUS	70°48.385′S	10°39.718′W	436	4 | 1 | -	1 | - | 1	- | - | -	2 | - | -	7 | 1 | 1	1 | - | 1	- | - | -	1 | - | 1
BX	70°56.348′S	10°33.998′W	313	46 | 5 | -	16 |2 | 11	- | - | -	22 | 3 | 2	84 | 10 | 13	2 | - | 7	- | - | -	2 | - | 7
total				199 | 20 | 2	68 | 9 | 12	35 | 10 | -	24 | 3 | 2	326 | 42 | 16	25 | - | 25	- | - | 12	25 | - | 37

The number of available sequences (after successful amplification) are given for each genetic marker per genus and species. First values = ITS; second = 18S; third = COI. – indicates that no sequence could be obtained. Sequence numbers have been summed per species (‘total’), and per population (‘total per location’)

DESS samples were carefully screened under a stereomicroscope (50 × magnification) and individuals from both targeted genera were handpicked with a fine needle and washed in three separate dishes with sterile distilled water to remove all remaining DESS compounds. Individuals were mounted on a temporary microscopic slide in a drop of distilled water and identified under a Leica DLMS compound microscope (1000 × magnification). During this ‘vouchering’ process, each specimen was assigned to a certain morphological group based on conspicuous body features, which were photographed at different magnifications. For *Sabatieria*, we distinguished three morphological groups, with differences in tail shape, number of amphid turns and male copulatory organs (see Additional file 1: Table S1.1). For *Desmodora* at least three distinct morphological groups (cf. *D. campbelli*, *D.* sp.A/B and *D.* sp.D of [32]; Additional file 1: Table S1.1) were recognised based on body length, position and length of somatic setae, male precloacal supplements and spicule apparatus, and presence of lateral body lines. After the vouchering process (5–10 min per specimen), each nematode was transferred individually into a microcentrifuge tube containing 20 μL Worm Lysis Buffer (WLB: 50 mM KCl, 10 mM Tris–HCl pH 8.3, 2.5 mM MgCl_2_, 0.45% NP40, 0.45% Tween 20; [45]), and stored at −20 °C.

### DNA extraction, amplification and sequencing

Proteinase K (1 μL; 10 mg mL^−1^) was added to the WLB-stored specimens for digestion after which samples were incubated at 65 °C for 1 h, followed by 10 min at 95 °C. They were centrifuged for 1 min at 14,000 rpm prior to usage of the DNA. Three markers were amplified by polymerase chain reaction (PCR): the nuclear ribosomal DNA (rDNA) Internal Transcribed Spacer (ITS) region, part of the mitochondrial cytochrome *c* oxidase subunit 1 (COI) gene, and for a subset of *Sabatieria* specimens, part of the nuclear small subunit (18S) rDNA. Details on the amplification process can be found in Additional files 2, 3, and 4 of the Supplementary Information. Sequences can be found in GenBank under accession numbers LT577954 – LT578168.

### DNA sequence alignments

Electropherograms of the COI, ITS and 18S sequences were analysed and assembled with lasergene® Version 7.1.0 (DNASTAR, Madison, WI) and trimmed to remove primer ends. Sequence length after trimming varied between 307 and 313 bp for COI of *Sabatieria*, 647–662 for COI of *Desmodora*, 647–662 for ITS of *Sabatieria*, 591–599 for ITS of *Desmodora*, and 845–864 bp for 18S of *Sabatieria*. Sequences were aligned for the two genera and each gene separately using clustalw v2 with default gap opening/extension costs of 15/6.66 in mega v6.0 [46, 47]. COI sequences were translated to amino acids using genetic translation Table 5 (invertebrate mitochondrial) to assign the correct reading frame and to verify that no indels or stop codons were present in the alignment. For each alignment, the best fit substitution model was selected in jModelTest [48, 49], using the Bayesian Information Criterion (BIC) (Additional file 1: Table S1.3).

### Phylogeny

The different alignments were analysed using different tree construction algorithms to inspect for the presence of concordant terminal clades among different markers. Maximum likelihood (ML) trees (bootstrap replication = 1000) were generated with RAxML v8.2.4 [50]. Ultrametric trees were produced using beauti v1.8.2 and beast v1.8.2 (Bayesian Evolutionary Analysis Sampling Trees; [51]) under different substitution models (Additional file 1: Table S1.3), lognormal relaxed clock model, and coalescent tree prior. A Markov Chain Monte Carlo analysis was run for 10 million generations, of which every 1000th generation was sampled, resulting in 10,000 Bayesian trees. Convergence of runs was checked in Tracer v1.6 [52], after which the first 5000 trees were discarded as burn-in, while the last 5000 trees were used to construct a consensus tree in TreeAnnotator v1.8.2 (beast package) and define posterior probabilities. Resulting consensus trees for all markers were visualised in FigTree (http://tree.bio.ed.ac.uk/software/figtree/) and used in further analysis. ML and beast analyses were run on the xsede server of the cipres Science Gateway v3.3 (https://www.phylo.org; [53]).

### DNA-based species delimitation

To test whether sequence datasets constituted a single or multiple species, a General Mixed Yule Coalescent (GMYC) model approach was applied [54]. Using the ultrametric gene tree as input, the GMYC algorithm compares two alternative models: i) a single coalescence model that assumes a single species, and ii) a model that combines a coalescent model of intraspecific branching with a Yule model for interspecific branching, thus assuming multiple species. The location of the switch (threshold T) from speciation to coalescence nodes is then fitted on the tree, resulting in an estimation of species diversity. Species delimitation under a single-threshold GMYC model was assessed in R [55] using packages ‘ape’ [56] and ‘splits’ [57]. Lineages-Through-Time (LTT) plots marking the position of threshold T on a relative timescale were constructed in R.

The presence of species-level lineages in sequence variation was also assessed by means of statistical parsimony [58]. tcs v1.21 software [59] partitioned the data into independent haplotype networks (gaps = missing data), connected by changes that are non-homoplastic with a probability of 95%. Final TCS haplotype networks [58, 60] were built using the PopART software (http://popart.otago.ac.nz), which only takes unambiguous sites into account.

We relied on a conservative consensus approach towards reconciling the results of the different species delimitation methods to maximise the reliability of species boundaries. More specifically, we recognised species clades that 1/received high nodal support (at least 75% bootstrap support in the ML tree), 2/showed compatible patterns based on statistical parsimony and GMYC analyses, 3/formed concordant clades in the trees inferred from nuclear and mitochondrial markers and/or expressed different morphological characteristics. Mean inter- and intraspecific differences (using a K2P (+ G) correction; Additional file 1: Table S1.3) were calculated in mega v6.0.

### Population genetics

Population genetic analyses were performed on ITS for *Sabatieria* and COI for *Desmodora* species as these were the most complete datasets (see later). Single-level Analysis of Molecular Variance (AMOVA; 1000 permutations, 0.05 significance level) was carried out in Arlequin v3.5.1.2 [61] to calculate fixation index *Φ*_st_ [62]. The different sampling sites were considered the different populations. Only species clades consisting of more than two populations with more than five individuals each were included in population genetic analyses. Standard measures of genetic variation within populations, such as nucleotide diversity (π; [63]) and gene diversity (*h*; [63, 64]) were also assessed in Arlequin. Intra-population and pairwise inter-population divergences were calculated where appropriate, using pairwise deletion of gaps and K2P-corrected distances (based on jModelTest results, Additional file 1: Table S1.3). Finally, isolation by distance (IBD) was assessed through Mantel testing in ibdws v3.23 [65] based on DNA sequences (ignoring gaps; between-population distance *Φ*_st_; between-sequence distance K2P) and 1000 randomisations.

## Results

### *Sabatieria*

#### Phylogeny

**Fig. 2:**
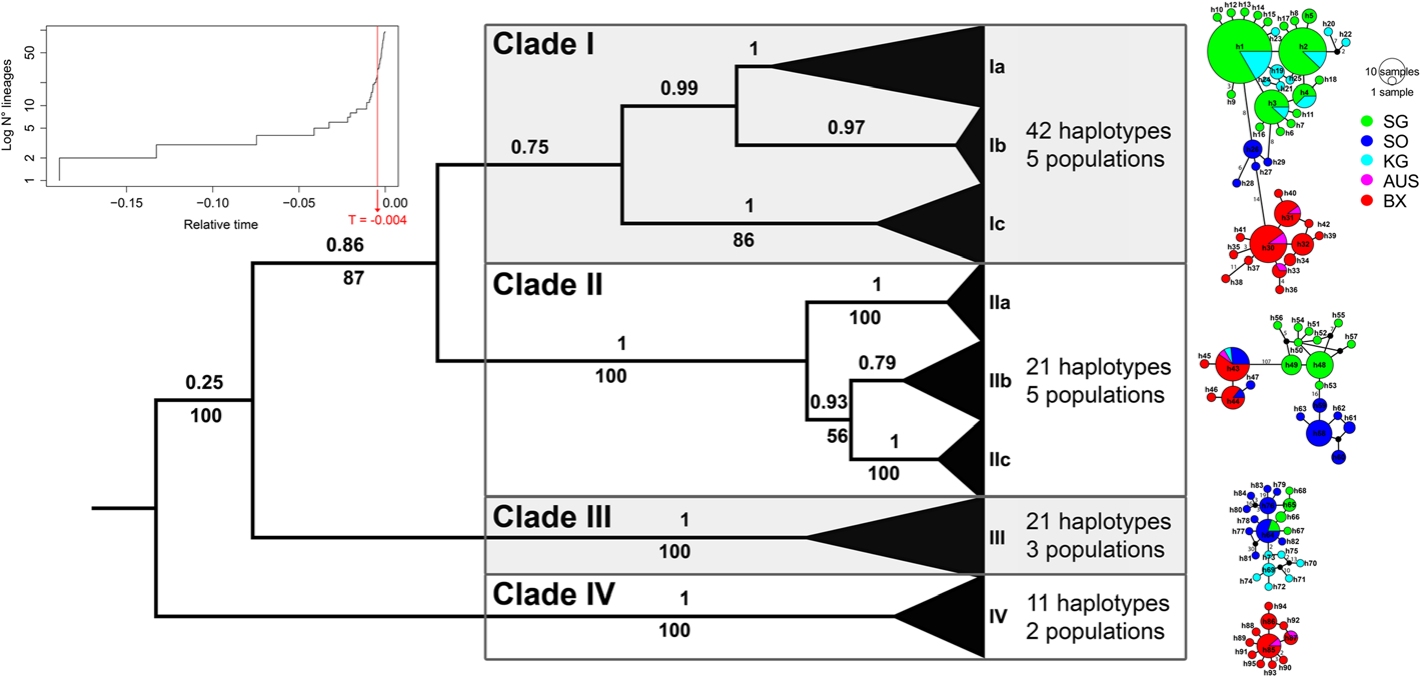
Phylogeny and population genetic haplotype networks for ITS of Sabatieria. Upper left corner: Log-lineages through time plot (LTT) indicating position of threshold time T (red line). Middle: Bayesian tree of ITS haplotypes of *Sabatieria*; numbers above branches indicate posterior probabilities, numbers below (where indicated) are ML bootstrap percentages (only when values >50%). Number of populations (i.e. geographical locations) and haplotypes are indicated next to each clade. Right: corresponding TCS haplotype networks of all four ITS clades for *Sabatieria*. Values along branches indicate the number of base pair differences between the two connecting haplotypes. When this number is not indicated, there was only 1 mutation. Black dots represent missing haplotypes. Size of circles is proportional to the amount of individuals belonging to that specific haplotype. Colour code based on the different locations

The alignment of 326 ITS rDNA sequences of *Sabatieria* was 679 sites long, containing 276 variable sites (196 parsimony informative) and 18 indel sites. Bayesian and maximum likelihood trees inferred from ITS haplotypes (see further) separated the sequences into four highly differentiated and relatively well-supported clades according to morphotype and/or geographic location (clades I – IV; Fig. 2). Individuals in clades I and II had the same physical appearance (morphological group 1; Additional file 1: Table S1.1), and were further divided into several sub-clades corresponding to different geographical locations (Ia – Ic, and IIa – IIc in clades I and II, respectively). Specimens belonging to clades III and IV were morphologically distinguishable (morphological group 2 and 3, respectively; Additional file 1: Table S1.1). Individuals in clade III had a different amphid and spicule shape, while individuals in clade IV had a blunt tail end (as opposed to the clavate tail tip typically observed in *Sabatieria*).

**Fig. 3:**
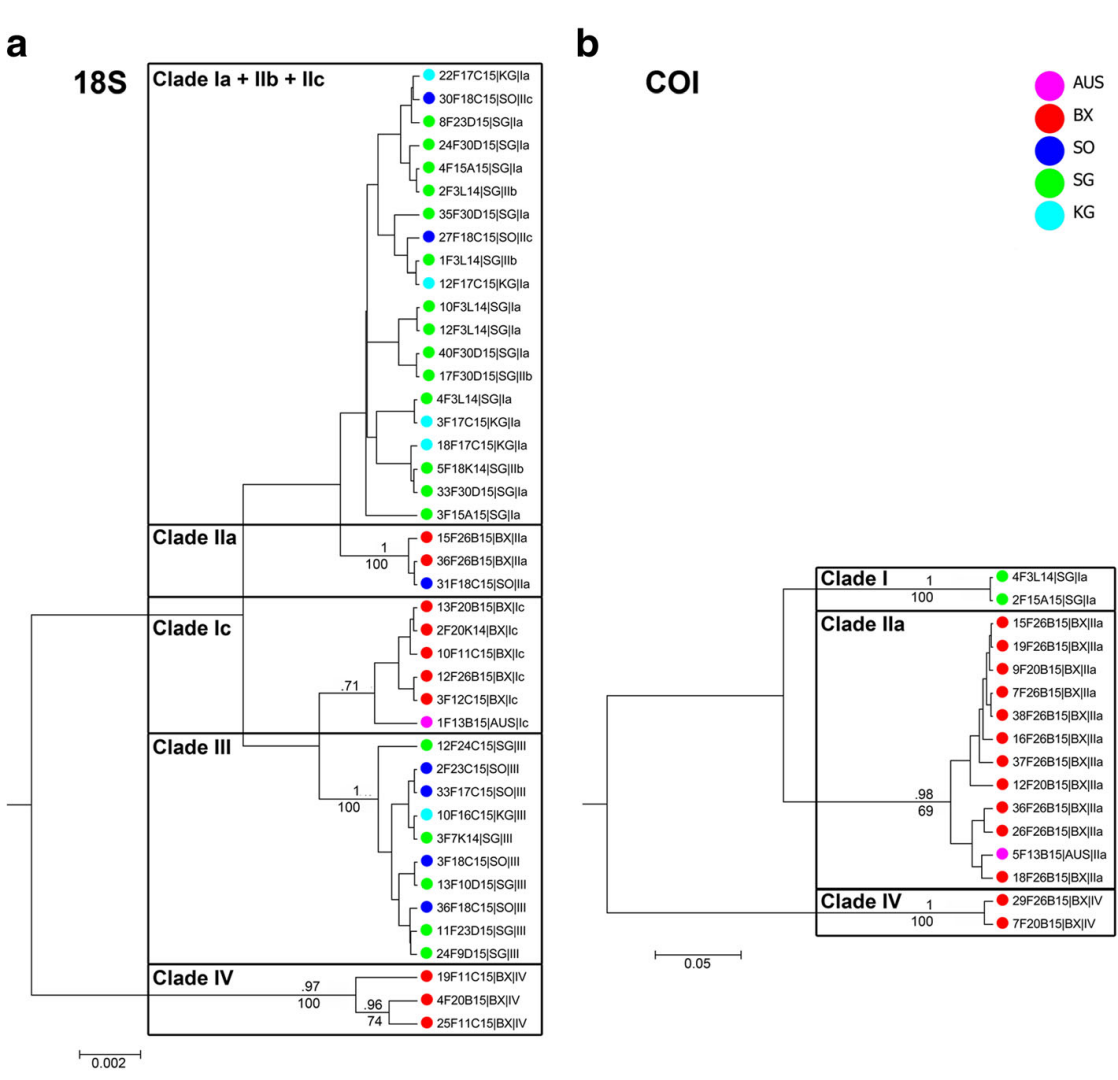
Bayesian trees for **a** 18S and **b** COI of *Sabatieria*. Numbers above branches indicate posterior probabilities as calculated by beast procedure, while numbers below branches depict ML bootstrap percentages from RAxML files. Only values above 50 are included in the graphs. Scale length represents number of substitutions per site. Colours represent location

Phylogenetic results based on ITS haplotype sequence data were compared with those based on a subset of the slower-evolving 18S rDNA (*n* = 42, alignment length 864 bp, 47 variable sites, 30 parsimony informative; Fig. 3a), and an unlinked similarly variable mitochondrial marker (COI; *n* = 16, alignment length 313 bp, 120 variable sites, 113 parsimony informative; Fig. 3b). In both cases, the phylogenies were generally congruent with the ITS tree, although not all ITS clades had COI sequence representatives due to amplification difficulties (see Additional file 2). The 18S tree did include individuals of all ITS clades, and showed high nodal support for clades IIa, III and IV (Bayesian posterior probabilities >.95; ML bootstrap values 100; Fig. 3a). The rest of the sequences were lumped into two clades with low support (Ia + IIb + IIc and Ic). COI sequence data showed high support for clades I and IV with posterior probabilities and ML bootstrap values of (almost) 100, and also clade IIa specimens formed a (less well-supported) clade (Fig. 3b). Hence, despite less successful amplification of COI and 18S data for *Sabatieria*, some of the same clades were recovered in tree topologies.

#### DNA-based species delimitation

Statistical parsimony analysis collapsed the 326 ITS sequences into 95 haplotypes (sequence divergence based on K2P distances = 0.2–26%) and 7 separate haplotype networks (Ia/b, Ic, IIa, IIb, IIc, III and IV; connection limit = 95% or 11 mutations), all corresponding to clades or sub-clades of the Bayesian tree (Fig. 2). The GMYC model gave a significantly better fit for the ITS data (likelihood ratio = 20.6; *P* < 0.001) than did the null model assuming uniform branching rates. The position of the threshold time T, marking the transition from between- to within-species rate of lineage branching, was estimated at −0.004 on a relative timescale (Fig. 2 upper left), and coincided with 21 species clusters. The confidence interval for the estimated number of species ranged from 8 to 26. As opposed to ITS, the GMYC model was insignificant when applied to 18S and COI data (*P* > 0.1), possibly as a consequence of the low number of sequences available.

Based on the three pre-defined criteria for species delimitation, the *Sabatieria* ITS dataset was divided into 4 putative species (clades in Figs 2, 3): 1/statistical parsimony and GMYC outcome pointed towards the presence of several species (note that GMYC indicated many more species clusters, but these were not supported by the other criteria); 2/nodal support in the ML tree topology for the four clades was substantial; 3/unlinked loci (ITS & COI) consistently recovered species I, II and IV, while species III (for which COI data is lacking) was considered a valid species based on its morphological differences with the other three species. The level of sequence divergence between the four species (average K2P distances between 11 and 21%) was considerably higher than within-species distances (~ 0.2 to 4%) (Table 2), giving further indication for species-level divergence. Also for 18S and COI, sequence divergence within putative species was distinctly lower than between species (especially for COI; Table 2).

**Table 2:** Mean intra- and interspecific genetic divergence for *Sabatieria*

	species I	species II	species III	species IV
*ITS Sabatieria (n = 326; 679 bp)*				
species I	1.40 ± 0.28			
species II	11.09 ± 1.24	3.73 ± 0.50		
species III	15.16 ± 1.59	20.71 ± 1.93	1.26 ± 0.15	
species IV	14.92 ± 1.60	18.56 ± 1.73	19.86 ± 1.90	0.22 ± 0.08
*18S Sabatieria (n = 42; 864 bp)*				
species I	0.15 ± 0.05			
species II	0.24 ± 0.08	0.24 ± 0.11		
species III	1.57 ± 0.41	1.70 ± 0.41	0.22 ± 0.09	
species IV	1.48 ± 0.33	1.58 ± 0.34	2.87 ± 0.52	1.13 ± 0.28
*COI Sabatieria (n = 16; 313 bp)*				
species I	0.00 ± 0.00			
species II	25.20 ± 3.24	1.49 ± 0.38		
species III	–	–	–	
species IV	37.78 ± 4.37	37.09 ± 4.14	–	0.64 ± 0.45

Values are K2P distances (gamma = 4 for ITS and COI; uniform rates for 18S) and are given in percentages with their standard error. Diagonal values are intraspecific divergences, while values below diagonal represent interspecific divergences. n = number of individuals analysed. – no data available

#### Population genetics

Of the four *Sabatieria* species recognised, three were used in population genetic analyses (I – III). Species I and II were clearly the most abundant (*n* = 200 and 66 ITS sequences, respectively), genetically diverse (42 and 21 ITS haplotypes, respectively) and widespread, comprising populations from both sides of the Weddell Sea (Fig. 2; Additional file 1: Table S1.4). Single-level AMOVA (Table 3) yielded large and significant among-population differences for both species (*Φ*_st_ = 0.886 and 0.765; *P* < 0.001), as could already be suspected from tree topologies (cf. sub-clades Ia – Ic; IIa – IIc) and haplotype networks (Fig. 2). Pairwise *Φ*_st_ values (Table 4) between populations of species I were significant in all cases except between AUS and BX (clade Ic), and between KG and SG (clade Ia). Most haplotypes were limited to one location, but in case they were shared (7 haplotypes), it was always between neighbouring locations at one side of the Weddell Sea (Additional file 1: Table S1.4). Average K2P divergence ranged between 0.23 and 3.28% (Additional file 1: Table S1.5), and was higher between populations on both sides of the Weddell Sea (e.g., BX and SG) than between populations on either side. Pairwise comparisons for species II were always significant, and again larger for populations divided by the Weddell Sea (SG vs. BX, SO vs. BX) than at the same side of it (SG vs. SO). As for species I, almost all haplotypes were restricted to a particular location, except for two that were shared among locations at both sides of the Weddell Sea (Additional file 1: Table S1.4).

**Table 3:** Single-level AMOVA results for each *Sabatieria* species based on ITS sequence data

Source of variation	df	var (%)	*Φ*_st_	*P*
***Species I***				
Among populations	4	88.59	**0.886**	***
Within populations	195	11.41		
***Species II***				
Among populations	2	76.48	**0.765**	***
Within populations	63	23.52		
***Species III***				
Among populations	2	17.84	**0.178**	***
Within populations	32	82.16		

Values are based on a K2P model, as indicated by jModelTest. df = degrees of freedom, var. = percentage of variation, *Φ*_st_ = fixation index, *P* = permutational P-value, based on 1000 permutations. Significant *Φ*_st_ values are indicated in bold. Significance codes: *** *P* < 0.001

**Table 4:** Pairwise *Φ*_st_ values between populations of the different *Sabatieria* species based on ITS sequence data

***Species I (n = 200)***	**SG** (114)	**SO**	**KG**	**AUS**
**SO** (8)	0.857 ***			
**KG** (27)	0.028 ^NS^	0.778 ***		
**AUS** (5)	0.938 ***	0.898 ***	0.896 ***	
**BX** (46)	0.927 ***	0.878 ***	0.898 ***	−0.098 ^NS^
***Species II (n = 66)***	**SG** (25)	**SO**		
**SO** (25)	0.597 ***			
**KG**	–	–		
**AUS**	–	–	–	
**BX** (16)	0.955 ***	0.743 ***	–	–
***Species III (n = 35)***	**SG** (8)	**SO**		
**SO** (19)	0.002 ^NS^			
**KG** (8)	0.380 ***	0.235 ***		
**AUS**	–	–	–	
**BX**	–	–	–	–

Numbers between brackets indicate the amount of individuals for each population. Species with only two populations (i.e. species IV) were not included and populations consisting of a single individual have not been taken into account. Significance codes: NS = non-significant, *** *P* < 0.001

Species III and IV were restricted to one side. Species III occurred at the western side of the Weddell Sea and consisted of three populations (SG, SO & KG) for which genetic structuring was significant, but considerably lower than for species I and II for the same populations on this side of the Weddell Sea (AMOVA *Φ*_st species III_ = 0.178, *P* < 0.001; Table 3; *Φ*_st species I & II_ = 0.589–0.599, *P* < 0.001; results not shown). Within-population variation for species III (~ 82%) exceeded that between populations (17.8%). Genetic differences were non-significant between locations SG and SO (Table 4), which also shared one haplotype (Additional file 1: Table S1.4). Average K2P distances between these populations were also clearly lower than for the other two species (Additional file 1: Table S1.5). Species IV was restricted to the two locations at the eastern Weddell Sea, and comprised 11 haplotypes. Within-population divergence was comparable or even larger than between-population differences, which were non-significant (Additional file 1: Table S1.5).

Despite the observation that main differences between populations of species were situated between different sides of the Weddell Sea (hence, at a large spatial scale), genetic divergence did not consistently decrease with increasing geographic distance (IBD r-values for species I, II and III were non-significant; *P* > 0.05; Additional file 1: Table S1.6).

### *Desmodora*

#### Phylogeny

**Fig. 4:**
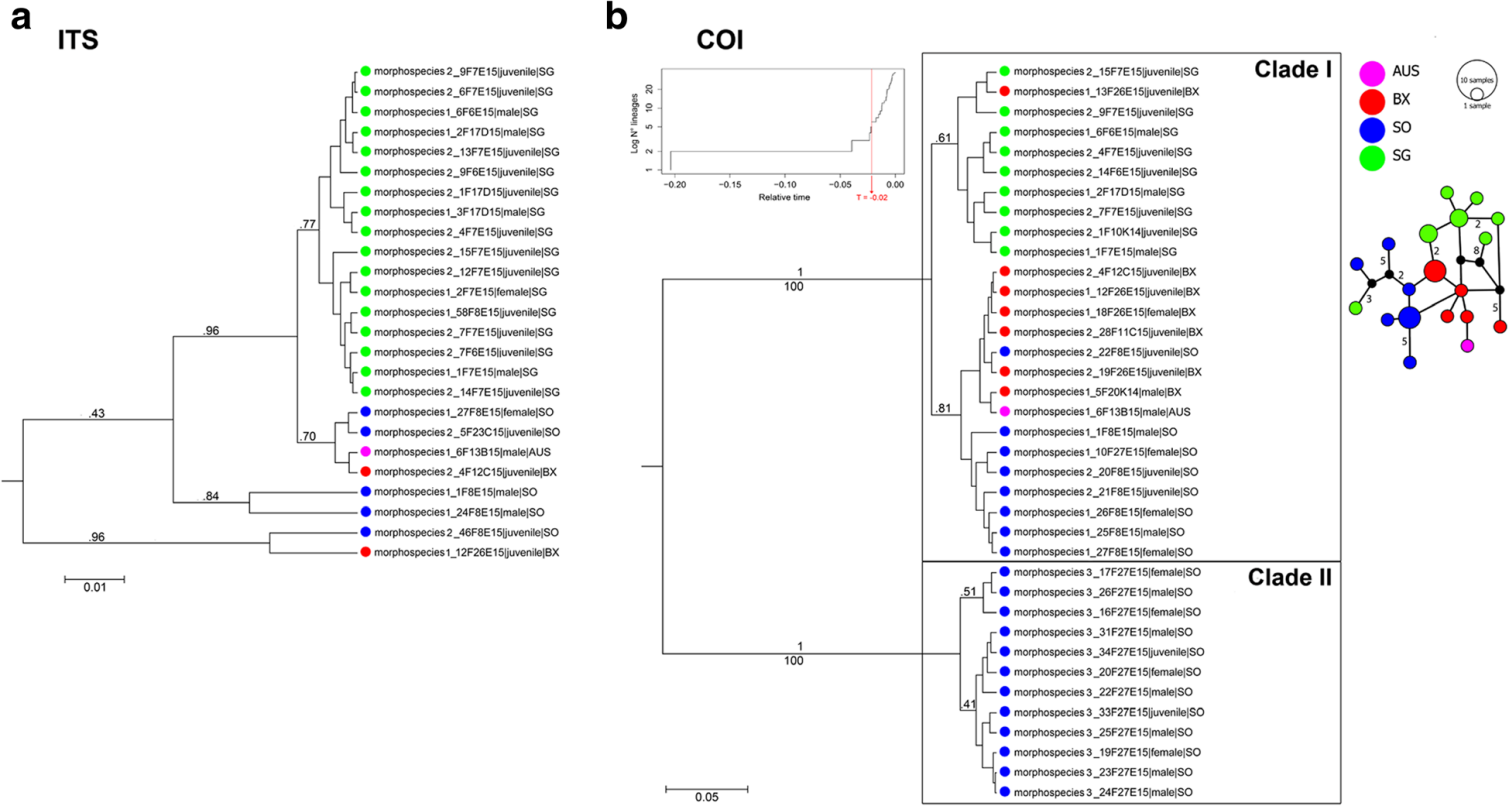
Bayesian trees of **a** ITS, and **b** COI haplotypes for *Desmodora*. Names of specimens are linked to morphological groups (here indicated as ‘morphospecies’). Numbers above branches are posterior probabilities; number below branches are maximum likelihood bootstrap values (only indicated when >50%). Colours represent locations. Scale length represents number of substitutions per site. The COI plot also includes the log-lineages through time plot with threshold time T indicated in red, and the haplotype network for *Desmodora* species I. Numbers along branches indicate the amount of mutations/base pair differences between the two connecting haplotypes. When this number is not indicated, there was only 1 mutation. Black dots represent missing haplotypes. Size of circles is proportional to the amount of individuals belonging to that specific haplotype. Colour code based on the different locations

The ITS alignment for *Desmodora* comprised 25 sequences and 599 sites, including 88 variable (41 parsimony informative) and 21 indel sites. For COI, the alignment included 37 sequences and 662 sites of which 196 variable (151 parsimony informative). *Desmodora* specimens showed distinct discontinuities in variation of several morphological features, including body size, amphid shape, male copulatory organs, and cuticle ornamentation (Additional file 1: Table S1.1). In contrast to *Sabatieria*, these morphological groups did not correspond to distinct clades in ITS tree topology (Fig. 4a). Most specimens were clustered irrespective of morphology, and both posterior probabilities and bootstrap values were low. In case posterior probabilities were above 0.95, bootstrap values were either very low (< 50), or specimens were not put into the same clade in the ML tree. As a result, it is highly unlikely that separate species lineages can be detected based on ITS data, and morphological differences between specimens are not diagnostic. In contrast to ITS, both Bayesian and ML tree topologies based on COI data hinted towards a clear differentiation between two species-level lineages (high posterior probabilities and bootstrap values), of which one corresponded to a different morphological group for which no ITS sequences were available (Fig. 4b; Additional file 1: Table S1.1). Further differentiation into sub-clades according to location as seen in the tree topology was never supported by high posterior probabilities and bootstrap values.

#### DNA-based species delimitation

The COI tree indicated two species lineages for *Desmodora* (clades I, II in Fig. 4), which was verified by the GMYC model (significant divergence: LR = 12.81, *P* < 0.01). The confidence interval for the number of species in the GMYC analysis was 2–7, but Bayesian posterior probabilities and ML bootstrap values clearly pointed towards the lower end of this range. Also statistical parsimony divided the COI data into two separate networks at the 95% probability level. Unfortunately, unsuccessful amplification of the ITS region of specimens belonging to clade II (= morphological group 3; Additional file 1: Table S1.1) prevented additional verification of this conclusion based on another unlinked genetic marker. However, co-occurrence of both species at the same location (SO), their high interspecific genetic divergence (Table 5) and morphological differences (Additional file 1: Table S1.1) strongly hint towards a separation into true species. They will therefore be considered as such in further analyses.

**Table 5:** Mean intra- and interspecific genetic divergence for COI of *Desmodora*

*COI Desmodora (n = 37; 662 bp)*	Species I	Species II
Species I	1.76 ± 0.25	
Species II	23.44 ± 2.08	1.59 ± 0.25

Values are K2P distances (gamma = 4). Diagonal values are intraspecific divergences with their standard error; value below diagonal is the interspecific divergence. n = number of individuals analysed

#### Population genetics

Population genetic structure within *Desmodora* was based on COI data (most complete dataset). Whereas species I occurred at both sides of the Weddell Sea, species II solely appeared in the South Orkneys samples (no population genetic structure to be tested). Genetic structuring between populations of *Desmodora* species I was significant, but lower than for *Sabatieria* species I and II (Table 6). Genetic variation within populations of *Desmodora* species I was comparable or sometimes even higher than between populations (Additional file 1: Table S1.5). A Mantel test for IBD within species I with three populations (SG, SO & BX) resulted also here in a non-significant r-value (*P* = 0.67; Additional file 1: Table S1.6), which is expected since dissimilarity is higher between populations SG and SO than between both of them and BX across the Weddell Sea (see Table 6).

**Table 6:** Single-level AMOVA main and pairwise results for *Desmodora* species I based on COI sequence data

**Source of variation**	**df**	**var (%)**	***Φ***_**st**_	***P***
***Species I***				
Among populations	2	26.55	**0.266**	***
Within populations	21	73.45		
**Pairwise** ***Φ***_**st**_ ***(n = 24)***	**SG** (9)	**SO**	**KG**	**AUS**
**SO** (8)	0.307 ***			
**KG**	–	–		
**AUS**	–	–	–	
**BX** (7)	0.286 ***	0.153 **	–	–

Values in brackets indicate the number of individuals per population. Populations of only one individual have not been taken into account. df = degrees of freedom, var. = percentage of variation, *Φ*_st_ = fixation index, *P* = permutational P-value, based on 1000 permutations. Significant *Φ*_st_ values are indicated in bold. Significance codes: ** *P* < 0.01, *** *P* < 0.001. n = number of specimens

## Discussion

### Conflict between morphological and phylogenetic species definitions in *Sabatieria* and *Desmodora*

Objective species delimitation is challenging in animal groups where taxonomic information is incomplete and scattered, yet remains fundamental in biodiversity research [5]. For this reason, a combination of several techniques and a conservative method were adopted to delineate species in this study. Congruence in the outcomes of various species delimitation approaches led to the recognition of four species-level lineages for *Sabatieria* and two for *Desmodora*. Not all of these coincided with the initial morphologically defined groups, and vice versa (Additional file 1: Table S1.1). In fact, rates of phenotypic and molecular divergence do not always converge [66], which makes species delimitation all the more tricky. Especially for relatively young species there might be an offset between the process of speciation and the acquisition of secondary properties such as distinct morphology. However, sequence divergence for COI in both genera was substantial (*Sabatieria*: 25–38%; *Desmodora*: 23%; Tables 2, 5; Figs. 3b, 4b), making the possibility of dealing with recent speciation less likely in this case. Within the genus *Sabatieria*, two out of four species differed from the others in morphological appearance (species III and IV), while the other two (species I and II) were not readily distinguishable and might constitute cryptic species. Cryptic speciation is common in marine free-living nematode genera (see [11] and references therein) and has also been recovered in limno-terrestrial nematodes on the Antarctic continent [67]. Also in other Southern Ocean benthic inhabitants, recent molecular findings have indicated that species which were previously considered eurybathic and/or circum-Antarctic can in fact be partitioned into cryptic species according to depth or geography [22, 25, 68]. Such a pattern has been observed among a variety of benthic invertebrates (e.g., amphipods, isopods, bivalves, crinoids and octopods), indicating that it is a common phenomenon among Southern Ocean species, and may result from isolation of smaller populations undergoing genetic bottlenecks in shelf or slope refugia during glacial times (see [25] for an overview). Especially for species with low dispersal capacity (such as nematodes), recolonisation of the continental shelf can be a slow process, possibly leading to (cryptic) speciation before secondary contact between previously isolated populations occurs. In contrast to *Sabatieria*, *Desmodora* specimens showed no evidence of cryptic speciation. Instead, the opposite phenomenon was observed where (conspicuous) morphological characteristics were not diagnostic in the delimitation of species. This observation of high intraspecific morphological variation for *Desmodora* casts doubt on previous reports of six different species within the genus based on morphological data for the same locations [32]. Similar high levels of intraspecific variation in morphology have been reported in the deep-sea nematode genus *Acantholaimus* from the Pacific [69], and in a *Paracanthonchus* species rafting on seaweed along the Brazilian coast [30]. In the latter study, these intraspecific morphological differences occurred in the absence of genetic differentiation among its populations. Some nematodes are even capable of resource polyphenism, a situation in which different phenotypes are induced by different thresholds of an environmental cue during their development [70, 71]. As such, relying on morphology alone when discriminating between nematode species may cause substantial bias.

### Wide and narrow species ranges in *Sabatieria* and *Desmodora*

This study showed that both *Sabatieria* and *Desmodora* contained species with wide ranges across the Weddel Sea, as well as species with more limited ranges on either side of the Weddell Sea. This combination of wide and narrow species ranges has been noted in several other Antarctic benthic invertebrates [72, 73], and these contrasting stories have been linked to the species’ dispersive capacities as well as different survival mechanisms during past glacial cycles [22]. *Sabatieria* species I and II (and also *Desmodora* species I) were distributed across locations separated by the deep Weddell Sea, indicating a connection at some point in time. Wide and even cosmopolitan species ranges have been reported in marine nematodes (e.g., [2, 9]) and can reflect ongoing dispersal as well as historical connections [74]. Given the fact that nematodes are passive dispersers and that locations in this study are separated by several hundreds of km, historical connectivity might be very important in this case (cf. [74, 75]). High levels of genetic divergence between species (Tables 2, 5) and long branches in tree topologies (Figs 3, 4) seem to support speciation in the distant past. On an evolutionary timescale, the origin of modern Antarctic biota is put shortly after the Gondwana break-up, which marked the onset of vicariance, speciation and diversification [76, 77]. Yet the resulting Antarctic Circumpolar Current (ACC) maintained a certain level of horizontal connectivity between species and populations along the continent, reflected in circum-Antarctic distributions observed in several benthic invertebrate species [22]. The large-scale distribution of both *Sabatieria* and *Desmodora* species might have a similar early origin of speciation followed by long-distance dispersal mediated by the presence of large current systems (ACC, ACoC, Weddell gyre) and relatively homogeneous environmental conditions (e.g., seabed temperatures) in the area [20, 78].

### High population genetic structure suggests low levels of gene flow in the Southern Ocean

The physical setting of the Southern Ocean – without obvious barriers to gene flow and with the presence of large-scale currents capable of mediating long-distance dispersal – did not change much over the course of history. Combined with the large population sizes of nematodes and the possibility of passive dispersal, this should result in mild genetic differentiation over large distances [79]. Nevertheless, population genetic structuring within *Sabatieria* and *Desmodora* species was substantial. Haplotypes were generally confined to a single geographic location or shared between neighbouring sites (only two *Sabatieria* haplotypes had representatives at both sides of the Weddell Sea; Fig. 2; Additional file 1: Table S1.4), a characteristic of closed populations and not uncommon in taxa that lack pelagic development [25, 74]. Pairwise *Φ*_st_ values for *Sabatieria* species I and II were significant in most cases and largest between locations at different sides of the Weddell Sea (Table 4). Similarly large genetic differences between eastern and western Weddell Sea were also revealed by COI and ITS sequences of benthic ostracods in the area [23]. *Desmodora* species I also showed highly significant pairwise *Φ*_st_ values (Table 6) but largest differences were situated between populations SG and SO, rather than between eastern and western Weddell Sea locations (Additional file 1: Table S1.5). These high levels of population genetic differentiation can have multiple origins. First, they might reflect poor dispersal capacity [25] and suggest that contemporary gene flow between populations is strongly limited at the spatial scale considered here. Similar studies for coastal and estuarine nematodes have demonstrated that population genetic structure can be significant at scales of 100 km and less [7, 8, 11], which is well below distances between the different locations for this study. If gene flow is indeed limited due to dispersal limitation, the large observed population genetic differences might point towards a limited efficiency of the ACC and Weddell gyre in homogenising nematode communities over large distances. Second, barriers to gene flow between populations in a marine setting can exist in many forms, such as temperature gradients, depth differences and large areas of unsuitable habitat conditions [11, 79]. The large pairwise differences between populations at both sides of the Weddell Sea and along the Scotia Arc might therefore result from such ‘invisible’ barriers to gene flow rather than true dispersal limitation. Finally, even in the presence of extensive dispersal between habitat patches, populations can show large genetic differences due to differences in the succesful establishment and reproduction of dispersers after settling in a new environment [80]. Local habitat conditions and species-specific niche preferences, followed by rapid adaptation and population growth may result in situations where priority effects, founder effects and genetic bottlenecks result in certain haplotypes being favoured over others [8]. However, such processes are generally assumed to be of less importance at large spatial scales [11].

### Phylogeographic patterns across the Weddell Sea do not support isolation by distance

Strong population genetic structure at large spatial scales (> 300 km) has been observed in many marine species [11, 75, 81], and has often been attributed to an isolation-by-distance mode of genetic differentiation. Yet for all species of *Sabatieria* and *Desmodora* with sufficient sample size, no IBD was observed (Additional file 1: Table S1.6). The reason for this is probably related to large variability in genetic divergence between Antarctic Peninsula and Scotia Arc populations. For example, in *Sabatieria* species I, gene flow was not restricted between populations SG and KG, located approximately 1600 km apart (non-significant small genetic distance; Table 4, Additional file 1: Table S1.5) but was very much so between SG and SO, which are separated by 900 km distance. This pattern was reversed in species III, where pairwise genetic differences between SG and SO were non-significant (Table 4, Additional file 1: Table S1.5). Within *Desmodora* species I, genetic differences were larger between SG and SO than between either of them and location BX at the other side of the Weddell Sea. Although it has been argued that the tip of the Antarctic Peninsula and Scotia Arc are highly connected due to the ACC system (e.g., [68]), our population genetic results do not support this. Instead, there seems to be a rather random pattern of genetic structuring between populations at the western Weddell Sea. Genetic structuring in other marine nematodes has shown such a chaotic pattern [11], which may be linked to oceanographic currents or other environmental variables [82] posing a certain level of biotic or habitat filtering on dispersing or settling individuals. For example, smaller-scale bottom currents and dynamics might hamper successful settlement, thereby decoupling dispersal from geographic distance (although mainly tested for species with pelagic larvae; [82]). Such current dynamic data were not assessed at the time of sampling, but could provide an explanation for the random genetic structuring in the stations near the Peninsula. In terms of local environmental conditions for the study locations, these were strongly correlated with geographic distance since largest discrepancies were noted between locations at both sides of the Weddell Sea. AUS and BX clearly had colder bottom temperatures (almost – 2 °C), lower amount of fresh food (assessed as chlorophyll a concentration in the sediment) and coarser sediment than the other three locations in the vicinity of the Antarctic Peninsula (Hauquier, personal observations). Hence, for the study area considered here, measured abiotic variables do not explain the observations of larger population genetic differences between closely located sites. Other variables (e.g., oxygen content) may provide additional explanations, but have not been assessed at the time of sampling. In any case, a better understanding of the complex interactions between species-specific life history traits on one hand, and habitat characteristics and hydrodynamics on the other hand may help to understand the highly variable dispersal patterns through space and time (see review by [83]), and the substantial patchiness observed in nematode community composition [11].

### Gene flow in the Weddell Sea is strongly reduced in both genera, but more so in the deeper sediment dwelling *Sabatieria* species

The two genera in this study share a similar endobenthic lifestyle, but population genetic structuring was more pronounced within the *Sabatieria* species than within *Desmodora* species I (cf. AMOVA results). This may be the result of their differential vertical distribution and feeding habits. Nematode dispersal is predominantly passive and mediated through hydrodynamic forces, but individuals living in sediment surface layers are more susceptible to resuspension and transportation in the water column, while deeper dwellers are rarely resuspended [36, 38, 84]. *Desmodora* prefers surface sediments where it can feed on algal particles scraped off the sediment grains, which potentially facilitated contemporary and historical gene flow over larger areas. Dispersal capacity of organisms plays an important role in connectivity between populations, and previous studies have indicated differences in structuring processes between active and passive dispersers (e.g., [75, 85]). Results of this study thus extend this knowledge and support the idea that vertical distribution in the sediment can be an important proxy for dispersal probability in marine nematodes (see also [2]).

## Conclusion

Our results demonstrate the occurrence of cryptic speciation in Antarctic continental shelf nematodes, and provide evidence for different mechanisms underlying spatial genetic structure within surface- and deeper-sediment dwelling nematode taxa. Historically, ocean current systems such as the ACC and Weddell gyre in the area may have served as a transportation route for species across the Weddell Sea, mainly for taxa occurring in surface sediments such as *Desmodora,* which showed less geographic structure in its distribution than the *Sabatieria* species. Currently, dispersal limitation in marine nematodes effectively hampers large-scale connectivity between populations across the Weddell Sea. At a smaller spatial scale, population genetic structuring on the western side of the Weddell Sea is rather random.

## Additional files

Additional file 1: Additional tables [86–89]. (DOC 139 kb)

Additional file 2: Detailed information on DNA amplification protocol [90–94]. (DOC 33 kb)

Additional file 3: Alignment of 18S rDNA sequences used in ITS primer development (fasta format). (TXT 104 kb)

Additional file 4: Alignment of 28S rDNA sequences used in ITS primer development (fasta format). (TXT 31 kb)
