# Supplementary material for: Distinct genetic differentiation and species diversification within two marine nematodes with different habitat preference in Antarctic sediments
Source: BMC Evol Biol. 2017 May 30;17:120. doi: 10.1186/s12862-017-0968-1 (PMC5450352; doi:10.1186/s12862-017-0968-1)
Supplement: Supplementary file 1 — Additional tables [86–89]. (DOC 139 kb) [file 12862_2017_968_MOESM1_ESM.doc]

**Distinct genetic differentiation and species diversification within two marine nematodes with different habitat preference in Antarctic sediments**

Freija Hauquier^1*^, Frederik Leliaert^1,2^, Annelien Rigaux^1^, Sofie Derycke^1,3^, Ann Vanreusel^1^

^1^ Marine Biology Research Group, Biology Department, Ghent University, Krijgslaan 281, 9000 Ghent, Belgium, freija.hauquier@ugent.be annelien.rigaux@ugent.be, ann.vanreusel@ugent.be

^2^ Botanic Garden Meise, Nieuwelaan 38, 1860 Meise, Belgium, frederik.leliaert@gmail.com

^3^ Operational Directorate Taxonomy and Phylogeny, Royal Belgian Institute of Natural Sciences (RBINS), Rue Vautier 29, 1000 Brussels, Belgium, sofie.derycke@naturalsciences.be

**BMC Evolutionary Biology**

**APPENDIX S1.** Additional tables containing information on morphospecies (Table S1.1), GenBank comparison (Table S1.2), substitution models used (Table S1.3), haplotypes (Table S1.4), intra- and interspecific genetic differences (Table S1.5) and isolation-by-distance results (Table S1.6).

**Table S1.1. Overview of the morphological groups per genus, used in initial identification.**

| ***SABATIERIA*** | **amphid** | **tail shape** | **precloacal supplements** | **spicule shape** | **max body length** | **cephalic setae** |  |
| --- | --- | --- | --- | --- | --- | --- | --- |
| morphospecies 1 | 1.5 turns | clavate/ short | > 15 | clear S-shape | > 2.5 mm | 2 rows of stout setae |  |
| morphospecies 2 | 2.5 turns / large | clavate/elongate | 11-13 | short, broader towards end | 2.5 mm | 1 row of long setae |  |
| morphospecies 3 | 2 turns | blunt/stout | na | na | < 1.5 mm | 2 rows of stout setae |  |
| ***DESMODORA*** | **amphid** | **tail shape** | **precloacal supplements** | **spicule shape** | **max body length** | **somatic setae** | **lateral line** |
| morphospecies 1 *(campbelli)* | 1 turn / large | conical/short | present | short, broader towards end | 1.5 - 2 mm | long, all over body | very obvious |
| morphospecies 2 (sp.D) | 1 - 1.5 turns / small | conical/short | na | na | < 1.5 mm | short, all over body | obvious |
| morphospecies 3 (sp.A/B) | 1.5 turns / small | slender/elongate | absent | longer, slender | ~ 1 mm | inconspicuous/absent | absent |

For *Desmodora*, the three morphological groups correspond to the species described by [32]. For *Sabatieria*, morphospecies were assigned based on the characteristics provided here. na = not assessed

**Table S1.2. Summary of blastn results for *Sabatieria* (18S) and *Desmodora* (COI) species.**

| **GENBANK** |  | ***Sabatieria* % coverage** | | | | ***Sabatieria* % similarity** | | | |
| --- | --- | --- | --- | --- | --- | --- | --- | --- | --- |
| **species** | **accession** | **sp. I** | **sp. II** | **sp. III** | **sp. IV** | **sp. I** | **sp. II** | **sp. III** | **sp. IV** |
| *Sabatieria sp.* | AY854238 | 99 – 100 | 99 – 100 | 100 | 97 - 98 | 98 - 99 | 99 | 98 - 99 | 97 - 98 |
| *Sabatieria punctata* | AY854235 | 99 – 100 | 99 – 100 | 100 | 97 - 98 | 98 - 99 | 99 | 98 - 99 | 97 - 98 |
|  |  | ***Desmodora* % coverage** | | | | ***Desmodora* % similarity** | | | |
|  |  | **sp. I** | **sp. II** |  |  | **sp. I** | **sp. II** |  |  |
| *Metachromadora sp.* | KC014987 | 91 – 93 | 91 – 92 |  |  | 81 - 82 | 82 -83 |  |  |

**Table S1.3. Overview of jModelTest output [48,49] for the different genetic markers of both genera.**

| **genus** | **marker** | **1^st^** | **BIC** | **reference** | **2^nd^** | **BIC** | **reference** | **BEAST** | **distances** |
| --- | --- | --- | --- | --- | --- | --- | --- | --- | --- |
| ***Sabatieria*** | 18S | K2P | 3714.8 | [86] | HKY | 3730.1 | [87] | HKY | K2P |
|  | ITS | K2P + G | 9301.3 | [86] | HKY + G | 9312.4 | [87] | HKY + G | K2P + G |
|  | COI | HKY + I | 2297.3 | [87] | K2P + I | 2309.9 | [86] | HKY + I | K2P + I |
| ***Desmodora*** | ITS | SYM + G | 3991.6 | [88] | GTR + G | 3997.0 | [89] | GTR + G | K2P + G |
|  | COI | HKY + G | 5312.7 | [87] | K2P + G | 5461.0 | [86] | HKY + G | K2P + G |

The latter two columns indicate the used substitution model* in beast analysis, and when calculating genetic distances. Not all models are incorporated in the different software programs, so second choices were also used. K2P = Kimura 2 parameter model; HKY = Hasegawa, Kishino & Yano model; SYM = symmetrical model; GTR = generalised time reversible model; + G = gamma rate variation between sites; + I = invariable sites; BIC = Bayesian Information Criterion. * Selected substitution models differ in the number of substitution rate parameters and base frequencies. The Kimura-2-parameter model (K2P; [86]) and Hasegawa-Kishino-Yano model (HKY; [87]) each consider two substitution classes (one transition and one transversion rate), but base frequencies are equal in K2P and variable in HKY. Under the generalised time reversible model (GTR; [89]), there are six substitution rates and variable base frequencies.

**Table S1.4. Unique and shared haplotypes, gene diversity and nucleotide diversity per population and species.**

| ***SABATIERIA*** |  |  |  |  |  |
| --- | --- | --- | --- | --- | --- |
| **Species I** | **unique haplotypes** | **shared haplotypes** | **number of individuals** | **gene diversity (h)** | **nucleotide diversity (π)** |
| **SG** | 14 | 4 | 114 | 0.731 | 0.002 |
| **SO** | 4 | 0 | 8 | 0.643 | 0.003 |
| **KG** | 7 | 4 | 27 | 0.860 | 0.004 |
| **AUS** | 0 | 3 | 5 | 0.833 | 0.002 |
| **BX** | 10 | 3 | 46 | 0.795 | 0.003 |
| **Species II** |  |  |  |  |  |
| **SG** | 10 | 0 | 25 | 0.767 | 0.003 |
| **SO** | 7 | 1 | 25 | 0.807 | 0.030 |
| **KG** | 0 | 1 | 1 | ‒ | ‒ |
| **AUS** | 0 | 1 | 1 | ‒ | ‒ |
| **BX** | 3 | 1 | 16 | 0.617 | 0.001 |
| **Species III** |  |  |  |  |  |
| **SG** | 4 | 1 | 8 | 0.893 | 0.004 |
| **SO** | 9 | 1 | 19 | 0.842 | 0.016 |
| **KG** | 7 | 0 | 8 | 0.964 | 0.012 |
| **AUS** | ‒ | ‒ | ‒ | ‒ | ‒ |
| **BX** | ‒ | ‒ | ‒ | ‒ | ‒ |
| **Species IV** |  |  |  |  |  |
| **SG** | ‒ | ‒ | ‒ | ‒ | ‒ |
| **SO** | ‒ | ‒ | ‒ | ‒ | ‒ |
| **KG** | ‒ | ‒ | ‒ | ‒ | ‒ |
| **AUS** | 0 | 2 | 2 | 1.000 | 0.002 |
| **BX** | 9 | 2 | 22 | 0.849 | 0.002 |
|  |  |  |  |  |  |
| ***DESMODORA*** |  |  |  |  |  |
| **Species I** |  |  |  |  |  |
| **SG** | 9 | 0 | 9 | 1.000 | 0.015 |
| **SO** | 8 | 0 | 8 | 1.000 | 0.015 |
| **KG** | ‒ | ‒ | ‒ | ‒ | ‒ |
| **AUS** | 1 | 0 | 1 | ‒ | ‒ |
| **BX** | 7 | 0 | 7 | 1.000 | 0.009 |

For Sabatieria values are based on ITS, and for Desmodora on COI data. Populations consisting of only one individual were not included in further analyses.

**Table S1.5.** **Mean intra- and interpopulation genetic divergences for the four Sabatieria ITS species and Desmodora COI species I.**

| ***SABATIERIA*** |  |  |  |  |  |
| --- | --- | --- | --- | --- | --- |
| **Species I (n = 200)** | **SG** | **SO** | **KG** | **AUS** | **BX** |
| **SG** | 0.17 ± 0.08 |  |  |  |  |
| **SO** | 1.49 ± 0.44 | 0.31 ± 0.10 |  |  |  |
| **KG** | 0.24 ± 0.09 | 1.57 ±0.45 | 0.31 ± 0.11 |  |  |
| **AUS** | 3.16 ± 0.68 | 2.53 ± 0.60 | 3.25 ± 0.68 | 0.15 ± 0.10 |  |
| **BX** | 3.19 ± 0.67 | 2.57 ± 0.59 | 3.28 ± 0.67 | 0.23 ± 0.08 | 0.32 ± 0.09 |
| **Species II (n = 66)** |  |  |  |  |  |
| **SG** | 0.34 ± 0.10 |  |  |  |  |
| **SO** | 6.20 ± 0.72 | 6.62 ± 0.65 |  |  |  |
| **KG** | ‒ | ‒ | ‒ |  |  |
| **AUS** | ‒ | ‒ | ‒ | ‒ |  |
| **BX** | 18.84 ± 1.92 | 15.63 ± 1.56 | ‒ | ‒ | 0.16 ± 0.08 |
| **Species III (n = 35)** |  |  |  |  |  |
| **SG** | 0.21 ± 0.11 |  |  |  |  |
| **SO** | 0.88 ± 0.14 | 1.42 ± 0.18 |  |  |  |
| **KG** | 1.15 ± 0.28 | 1.71 ± 0.28 | 1.18 ± 0.22 |  |  |
| **AUS** | ‒ | ‒ | ‒ | ‒ |  |
| **BX** | ‒ | ‒ | ‒ | ‒ | ‒ |
| **Species IV (n = 24)** |  |  |  |  |  |
| **SG** | ‒ |  |  |  |  |
| **SO** | ‒ | ‒ |  |  |  |
| **KG** | ‒ | ‒ | ‒ |  |  |
| **AUS** | ‒ | ‒ | ‒ | 0.15 ± 0.15 |  |
| **BX** | ‒ | ‒ | ‒ | 0.18 ± 0.09 | 0.23 ± 0.08 |
|  |  |  |  |  |  |
| ***DESMODORA*** |  |  |  |  |  |
| **Species I (n = 24)** |  |  |  |  |  |
| **SG** | 1.60 ± 0.26 |  |  |  |  |
| **SO** | 2.22 ± 0.37 | 1.49 ± 0.25 |  |  |  |
| **KG** | ‒ | ‒ | ‒ |  |  |
| **AUS** | ‒ | ‒ | ‒ | ‒ |  |
| **BX** | 1.80 ± 0.33 | 1.45 ± 0.27 | ‒ | ‒ | 0.95 ± 0.21 |

Values are based on K2P distances (gamma = 4). Populations of only one individual were not taken into account. Values are given in percentages with their standard error. Diagonal values are intra-population divergences, while values below diagonal represent interpopulation divergences. n = number of individuals analysed.

**Table S1.6.** **Isolation-by-distance Mantel test results for *Sabatieria* sp. I, II and III, and *Desmodora* sp. I.**

| **species** | **n** | **Z** | **r** | ***P*** | **intercept** | **slope** |
| --- | --- | --- | --- | --- | --- | --- |
| ***Sabatieria* sp. I** | 5 | 12968.28 | 0.62 | 0.15 | ‒ 0.10 | 0.00051 |
| ***Sabatieria* sp. II** | 3 | 3934.48 | 0.99 | 0.16 | 0.06 | 0.00040 |
| ***Sabatieria* sp. III** | 3 | 830.49 | 0.76 | 0.31 | ‒ 0.27 | 0.00044 |
| ***Desmodora* sp. I** | 3 | 1170.87 | ‒ 0.38 | 0.67 | 0.44 | ‒ 0.00012 |

n = number of populations, Z = test statistic, r = correlation coefficient, *P* = permutational P-value based on 1,000 randomisations, intercept = intercept of linear regression model, slope = slope of linear regression model.
