# Supplementary material for: Distinct genetic differentiation and species diversification within two marine nematodes with different habitat preference in Antarctic sediments
Source: BMC Evol Biol. 2017 May 30;17:120. doi: 10.1186/s12862-017-0968-1 (PMC5450352; doi:10.1186/s12862-017-0968-1)
Supplement: Supplementary file 2 — Detailed information on DNA amplification protocol [90–94]. (DOC 33 kb) [file 12862_2017_968_MOESM2_ESM.doc]

**Distinct genetic differentiation and species diversification within two marine nematodes with different habitat preference in Antarctic sediments**

Freija Hauquier^1*^, Frederik Leliaert^1,2^, Annelien Rigaux^1^, Sofie Derycke^1,3^, Ann Vanreusel^1^

^1^ Marine Biology Research Group, Biology Department, Ghent University, Krijgslaan 281, 9000 Ghent, Belgium, freija.hauquier@ugent.be annelien.rigaux@ugent.be, ann.vanreusel@ugent.be

^2^ Botanic Garden Meise, Nieuwelaan 38, 1860 Meise, Belgium, frederik.leliaert@gmail.com

^3^ Operational Directorate Taxonomy and Phylogeny, Royal Belgian Institute of Natural Sciences (RBINS), Rue Vautier 29, 1000 Brussels, Belgium, sofie.derycke@naturalsciences.be

**BMC Evolutionary Biology**

**APPENDIX S2.** Detailed information on the DNA amplification protocol used for the different genetic markers.

DNA AMPLIFICATION

In all cases, for both genera and the different markers, final reaction volumes for PCR were 25 µL, containing 14.875 µL nuclease-free water, 0.125 µL TOPTAQ Polymerase (Qiagen®), 2.5 µL 10 × PCR buffer with 15 mM MgCl_2_, 2.5 µL coral load PCR buffer 10 ×, 2 µL MgCl_2_ 25 mM, 0.5 µL dNTP (deoxynucleotide triphosphate, 10 mM), 0.250 µL primer (at 25 µM; both forward and reverse) and 2 µL DNA template. Used primers were JB3 (5’-TTTTTTGGGCATCCTGAGGTTTAT-3’) and JB5GED (5’-AGCACCTAAACTTAAAACATARTGRAARTG-3’) for COI of *Sabatieria* [7], and universal primers CO1490F (5’-GGTCAACAAATCATAAAGATATTGG-3’) and CO2211R (5’-AATGAGAATATAAACTTCWGGRTG-3’) for COI of *Desmodora*. The first primer combination yields a DNA fragment of approximately 320 bp, while the latter one gives an amplicon of roughly 720 bp. Both fragments do not overlap. For amplification of the ITS region of both genera, a new set of primers was developed (forward 18S-1F: 5’-GTCGTAACAAGGTTTYCGTAGGTGAACC-3’; reverse 28S-R: 5’-CCTTGTTAGTTTCTTTTCCTCCGCC-3’), resulting in a fragment of ~ 700 bp, including ITS-1, 5.8S and ITS-2 regions. These new primers were designed using guidelines described in https://www.thermofisher.com/be/en/home/products-and-services/product-types/primers-oligos-nucleotides/invitrogen-custom-dna-oligos/primer-design-tools.html, based on 18S and 28S rDNA alignments including species of Comesomatidae (including *Sabatieria*), Desmodorinae (including *Desmodora*), and a selection of other nematode species. Both alignments, including taxon information and GenBank accession numbers are available in the supplementary files Appendix S3 and S4. Forward primer 18S-1F starts at position 1907 in the 18S alignment. Reverse primer 28S-R starts at position 49 in the 28S alignment. Finally, primer combination G18S4 (F: 5’-GCTTGTCTCAAAGATTAAGCC-3’) and 4R (R: 5’-GTATCTGATCGCCKTCGAWC-3’) was used for amplification of approximately 860 bp of the 18S region of a subset of *Sabatieria* specimens (ITS haplotypes). PCR conditions for COI were initial denaturation for 5 min at 94 °C, followed by denaturation at 94 °C for 30 s, annealing at 50 °C for 30 s and extension at 72 °C for 45 s repeated in 40 cycles, followed by a final extension for 10 min at 72 °C. For ITS, conditions were: 3 min at 94 °C, followed by 40 cycles of 1 min 94 °C, 1 min 55 °C and 1 min 30 s 72 °C, and finally 5 min of extension at 72 °C. PCR of the 18S region of *Sabatieria* started with an initial step of 5 min at 94 °C, then 40 cycles of 30 s at 94 °C, 30 s at 56 °C and 1 min at 72 °C, again followed by a final extension step at 72 °C for 10 min. All PCR reactions included both a positive and a negative control. Positive controls contained genomic DNA of a marine nematode belonging to a different order (*Litoditis marina* (Bastian, 1865) Sudhaus, 2011), while negative controls only contained the PCR mixture, without addition of DNA.

Quality of PCR products was checked on 1 % agarose gels (stain = 0.0003 % ethidium bromide; size marker = 2 kbp DNA Easy Ladder (Bioline®)). Sanger sequencing was performed by Macrogen sequencing service (Macrogen Inc, Europe) with forward primers (JB3, CO1490F and 18S-1F; 10 µM) for all PCR products, and with both forward and reverse primers for the individual haplotypes. Sequences were verified with a blastn 2.3.1 search against the GenBank non-redundant nucleotide collection (nr/nt) [90] (Table S1.2 in Appendix S1). Dubious sequences (i.e. no hit with nematodes or low similarity (< 70 %) and/or coverage in the case of COI and 18S (< 85 %)) and short fragments were removed. Sequencing success (i.e. percentage of useful sequences out of total specimens subjected to PCR reaction) was as high as 92.6 % for ITS in *Sabatieria*, but much lower in all other cases (41.2 % 18S *Sabatieria*, 9.1 % COI *Sabatieria*, 31.6 % ITS *Desmodora* and 48.7 % COI *Desmodora*). Possible reasons for low sequencing success in marine nematodes are numerous, including lack of homology between targeted DNA and existing primer combinations (e.g., [28,91]), high variability of molecular markers among different groups within the phylum Nematoda (especially for the COI gene; [3,7,8,28,92]), poor quality and/or quantity of DNA templates (e.g., due to the nematodes’ small size or long preservation; [93]) and the presence of non-nematode DNA acting as a competitive template for primer binding (since the nematodes are digested as a whole, including any genomic DNA of organisms present inside the digestive tract and/or on the cuticle; [93,94]).
